# A Dietary Plant Extract Formulation Helps Reduce Flea Populations in Cats: A Double-Blind Randomized Study

**DOI:** 10.3390/ph16020195

**Published:** 2023-01-28

**Authors:** Damien Banuls, Jessie Brun, Jean-Louis Blua, Marie Christine Cadiergues

**Affiliations:** 1 Department of Clinical Sciences, Université de Toulouse, ENVT, 31076 Toulouse, France; 2 Laboratoires BIODEVAS, ZA de l’Epine, 72460 Savigné-L’Evêque, France; 3 INFINITy, Université de Toulouse, Inserm, CNRS, UPS, 31059 Toulouse, France

**Keywords:** cat, dermatology, flea, insecticide, natural, plant

## Abstract

There is a growing demand for natural products to be used to control fleas in pets. A prospective, double-blind, randomized, placebo-controlled study evaluated the efficacy of the biological plant-based food supplement Bioticks^®^ (thyme, rosemary, lemon balm, fenugreek, wormwood, and lemongrass extracts) as a flea control product in naturally flea-infested cats with an indoor–outdoor lifestyle. Ten cats were used as placebo controls (group A). Ten other cats were fed the same daily diet but supplemented with Bioticks^®^ (group B). Fleas were counted by combing at D0 and D0 + 14 days, then one, two, three, four, and five months after the start of this study. No flea treatment was administered, and no environmental changes were made for six months prior to the start and throughout this study. The product was well-tolerated. The mean flea population in group B progressively and steadily decreased to reach 3.3 ± 2.1 at month five. At the same time and under similar maintenance conditions, the average flea population in group A remained stable (14.3 ± 2.5) until the fifth month. The percentages of efficacy (Abbott formula) in group B compared to group A was 27%, 20%, 52%, 66%, and 77%, respectively, at one, two, three, four, and five months after the start of this study.

## 1. Introduction

Siphonaptera (fleas) are an order of widespread, ectoparasitic, flightless insects that feed on mammals and, to a lesser extent, on birds. Many fleas are highly specific to their host. In companion animal medicine, species of the genus *Ctenocephalides* (family Pulicidae) are most prevalent, but the proportion of infested animals varies with the location and the time of year and depends on whether or not the animal is a stray. Prevalence is usually around 20% of dogs and cats, slightly higher in cats [1,2,3], although data on stray animals indicate that as many as 90% of them may host fleas [4]. In both dogs and cats, the species *Ctenocephalides felis felis* (Bouché, 1835) is by far the most prevalent, while more host-specific species, such as *Ctenocephalides canis* (Curtis, 1826), are scarcer [3,5].

Many Pulicidae are important vectors of human and animal diseases, mainly rickettsioses, but also other diseases. For example, the oriental rat flea *Xenopsylla cheopis* is a vector of both *Yersinia pestis* and *Rickettsia typhi* [6]. *Ctenocephalides felis* is a known vector of *Bartonella henselae* (causing cat-scratch disease) and *Rickettsia felis* (recently found to cause cases of typhus in humans) [7]. As far as animal diseases are concerned, *C. felis* is known to transmit *Dipylidium caninum* tapeworms and *Mycoplasma haemofelis* [6,8]. Concerns arise about the possibility of transmission of cat retrovirus Feline Leukaemia Virus (FeLV) [9]. Flea-allergy dermatitis is a major allergic disease in both cats and dogs and is a hypersensitivity phenomenon linked to components of flea saliva [10,11].

Flea control is thus an important part of animal healthcare. In France in 2020, insecticides, repellents, and endectocides accounted for about 20% of the market share of veterinary medicines, about as much as vaccines, making them the second most widely sold veterinary drugs [12]. Similar figures are reported in the United States [13]. However, in recent years, growing evidence has been found for antiparasitic resistance in fleas [13,14], and while the arrival of isoxazolines on the market temporarily reduced this concern, sustainable management of flea populations remains topical. A wide range of molecules and galenic forms exists, which work either as repellents or as disruptors of the flea life cycle by killing adults, larvae, or eggs or by preventing normal larval development. The development of novel medications containing as-yet-unused compounds is critical. Furthermore, there is a growing demand both by pet owners and veterinarians for natural products for two reasons: safety for animals and minimizing the risk to human health and the environment.

Many plant metabolites, including alkaloids, terpenoids, and flavonoids, have been shown to have insecticidal properties [15,16]. Many essential oils, especially their monoterpene components, exhibit a variety of biological activities against a wide spectrum of insect pests. They can adversely affect growth and reproduction rate, the behavior traits of insect pests, and act as contact insecticide, fumigant, repellent, and antifeeding agents [17,18].

Thyme (*Thymus vulgaris* L.) is a plant originating from the Mediterranean basin and is a sub-shrub from the Lamiaceae. This plant is generally used for food applications (salad, energy drinks) as well as for humans and animals or as an agricultural companion plant to protect crops from insects. Thyme essential oil, in particular, is known to repel malaria, filarial, and yellow fever vectors [19], but only one in vitro study demonstrated the efficacy of Brazilian peppertree oil extract *Schinus molle* against adult fleas only [20]. Thymol, along with carvacrol and linalool, has shown potential toxic and repellent in vitro activity against castor bean tick Ixodes ricinus [21].

Rosemary (*Salvia rosmarinus* Spenn.) is a small evergreen plant of the Lamiaceae family. Native to the Mediterranean region, rosemary has naturalized throughout much of Europe and is widely grown in gardens in warm climates. The leaves are generally used to season foods but also to protect crops against insects. Rosemary has been reported to have antibacterial, antidiabetic, anti-inflammatory, antitumor, antioxidant, antinociceptive, and analgesic properties, among others [22]. Rosemary essential oil has been successfully tested against two-spotted spider mites on greenhouse tomato [23].

Melissa (*Melissa officinalis* L.) is a perennial plant also of the mint family (Lamiaceae). This aromatic herb, also called balm gentle or lemon balm, is grown for its lemon-scented fragrant leaves. It is native to the Mediterranean region and Central Asia and has naturalized in parts of North America and elsewhere. It is widely cultivated in temperate climates as a culinary and medicinal herb used in traditional medicine around the world and as a garden ornamental. It contains mainly flavonoids, terpenoids, phenolic acids, tannins, and essential oil. Antifungal, antioxidant, antidiabetic, antibacterial, and antimicrobial effects have been reported [24].

Fenugreek (*Trigonella foenum-graecum* L.) belongs to the family Fabaceae. Widespread throughout the world, it is an ancient medicinal plant and traditional food. Fenugreek seeds contain tannins, polyphenols, alkaloids, steroids, and saponins and are known to help regulate glycemic levels. Fenugreek seed also contains flavonoids that provide anti-inflammatory and antinociceptive properties. Polysaccharides and volatile compounds are obtained in abundant amounts in fenugreek seed [25].

Absinthe wormwood (*Artemisia absinthium* L.) is a plant species of the Asteraceae family. It is a perennial, herbaceous plant covered with silvery white hairs and numerous oil glands. Essential oils or extracts from different parts of *Artemisia* ssp. have been characterized by antibacterial, antifungal, and antioxidant activities [26].

Lemongrass (*Cymbopogon citratus* (DC.) Stapf), also called sweet rush, is a species of oil grass in the family Poaceae and is often used in cooking. The essential oil is considered a potent anti-inflammatory and antifungal drugs [27]. It is also found in mosquito-repellent inventions [28].

A preparation containing plant extracts with repellent properties was developed by progressively combining several active ingredients until a formulation with satisfactory activity on Dermanyssus galinae, the poultry red mite. The mixture of biological hydroalcoholic extracts of thyme, rosemary, melissa, fenugreek, absinthe, and lemongrass, especially antioxidant flavonoids, terpenes, and sulfur heterosides, was considered the optimal mixture to obtain the best results and was used in laying hens since 2006 (Mitarom^®^, Biodevas Laboratories, unpublished data). The product was then adapted for use in the control of fleas in pets. A dietary supplement containing these specific plant extracts was shown to significantly reduce flea infestation in dogs [29].

The aim of the present study was to assess whether the use of such plant extracts could help control flea populations in cats. We aimed to select a situation with an initial high to very high parasite load at the beginning of this study as evidence that the environment would be propitious for the completion of the flea cycle. We can thus infer high parasite pressure and the presence of large numbers of immature fleas in the environment. This degree of infestation may lead to cutaneous symptoms and pruritus, especially in sensitive animals.

## 2. Results

Twenty cats of mixed or pure breeds belonging to three separate owners, aged from 5 months to 19 years, in good general health and living both inside and outside, were included in this study. One group of ten cats (site I), belonging to the same owner and living together, formed group A; six cats living at site II and four cats living at site III formed group B. All three sites are located in the same geographical region in France.

The mean age of cats in group A was 5.35 ± 5.67 years; in group B, the mean age was 6.48 ± 5.46 years. The mean weight in group A was 4.22 ± 0.80 kg, and in group B, 4.10 ± 0.77 kg. Group A comprised three male and seven female cats; group B comprised two male and eight female cats.

Cats in group A received the neutral diet. Cats in group B received the same feed supplemented with Bioticks^®^.

### 2.1. Tolerance

All the cats completed this study. Their owners noticed no adverse reaction to the feed, whether supplemented or not. Clinical examinations performed after the beginning of this study revealed no abnormalities.

Prolonged handling of the cats and on-site examinations meant biological testing for tolerance was not feasible.

### 2.2. Antiparasitic Activity

At the time of inclusion, between 11 and 32 fleas were found on each cat, with a mean of 14.5 ± 2.4 fleas per cat in group A (10 cats) and 21.7 ± 5.7 fleas per cat in group B (10 cats), i.e., high to very high infestation.

**Figure 1:**
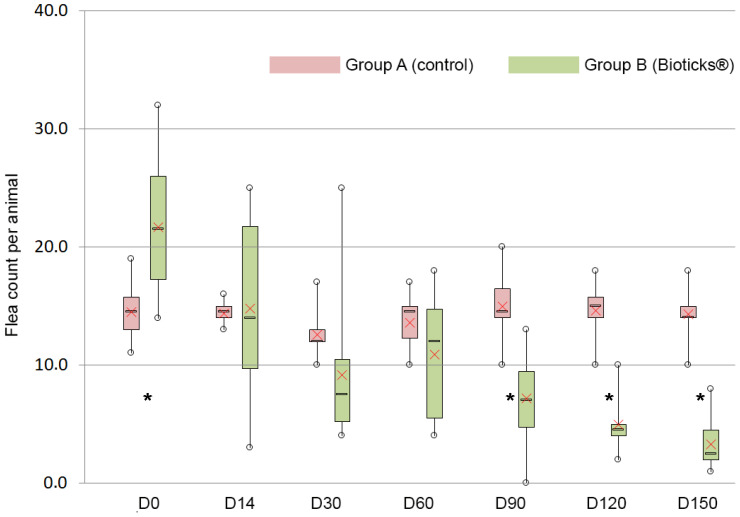
Distribution of the number of fleas per animal in each group according to the type of diet (red box = neutral, green box = supplemented) and to the time after the beginning of this study. In each box, the horizontal line represents the median value, the borders represent the 25th and 75th percentiles, and the whiskers show the range of all values. The red cross corresponds to the mean value. Stars * indicate a significant difference between groups (*p* ≤ 0.05).

In group A, the flea population remained fairly stable and was 14.3 ± 2.5 on D150. However, in group B, the number of fleas on cats housed in similar conditions consistently decreased, reaching 3.3 ± 2.1 on D150, i.e., low infestation (Table 1, Figure 1).

Infestation in group B was significantly higher at the beginning of this study; mean flea counts then remained comparable between D14 and D60. From D90 on, the difference between the two groups became statistically significant, with group A (control) harboring significantly more fleas than group B (treatment) at every examination.

### 2.3. Dermatological Examination

Dermatological examination revealed satisfactory skin and coat quality, with no significant dermatological lesions at any time in any cat.

Pruritus was observed in all cats in group A. One cat showed signs of pruritus as early as D60; four started showing pruritus on D90, four others on D120, and one on D150. In all cats except one, pruritus remained from its onset to the end of this study, even though its intensity did not increase consistently.

## 3. Discussion

In this study, the dietary supplement formulated for companion animals and made from a variety of hydroalcoholic plant extracts rich in flavonoids and terpenes was shown to have a significant capacity to reduce the parasite load of cats.

The use of plant extracts containing terpenes and flavonoids as novel antiparasitic compounds for companion animals is promising, at least in certain contexts. Flavonoids consist of two aromatic cycles and one oxygenated cycle, while terpenes are derived from the polymerization of isoprene. Both families can be widely substituted or isomerized, leading to a variety of secondary metabolites with numerous properties. Flavonoids are known to reduce both abiotic and biotic stresses in plants [16,30] and have been shown to have a deterrent effect on the feeding behavior of arthropods [31,32]. Terpenoids also play protective roles in plants, including against arthropod pests [15,33]. These compounds are of growing interest in both human and veterinary medicine, and their variety suggests a wide range of potential uses [34,35,36].

The product reached 77% efficacy vs. the placebo after five months of use, and the level of infestation of the treated group became low. Such infestation levels are generally considered tolerable for these animals, although they may still represent a reservoir. In our study, the cats in group B at both sites II and III showed no sign of pruritus, whereas cats in group A started showing signs of pruritus after a few months of high infestation. However, a low parasite load may be sufficient for the onset of symptoms of flea-allergy dermatitis [6,11], although the number of bites required to trigger obvious signs remains unknown and is likely to vary with the individual [10]. Moreover, continuous exposure to fleas has been shown to protect cats against becoming hypersensitive to flea saliva [11]. Reducing the parasite load is also a step forward in managing flea-borne pathogens, especially since many of these diseases require either ingestion or contact with a wound containing flea feces or fragments [8,37,38].

Even though the product tested in this study is not a drug and, as such, is not subject to the same evaluations, we sought to assess its efficacy according to the requirements of the EMA for antiparasitic drugs intended for pets [39]. Efficacy versus placebo already reached 50% after three months of continual use and peaked at 77% at the end of this study. This is below the 95% required by the EMA and, indeed, less than the currently available drugs [40].

The effect observed in this study only became noticeable after a few months. This could have several explanations based on proposed mechanisms of action inferred from existing products. Bioavailability was not measured, and the incorporation of sufficient amounts of compounds in skin sebum could take several weeks before showing repellent properties. Similarly to the antifeeding effect these metabolites have on insects when they circulate in sap, they may also deter parasites from feeding on the treated animal’s blood, thus reducing the duration of the blood meal [31]. This may create suboptimal conditions for the full completion of the flea’s life cycle, for example, fewer viable eggs. A direct effect on eggs and larvae in the environment is unlikely, which could explain in our study the longer time needed to efficiently reduce infestation pressure.

A recent study conducted on 22 adult hunting dogs using the same product similarly reduced flea infestation [29]. The protocol was similar to that used in the present study, except that all the dogs lived at the same site in semi-open kennels. Although they lived at the same site, there was no direct or indirect contact between the two groups for the duration of this study. The dogs in both groups started with a medium degree of infestation. Significant differences in flea counts started to appear after one month of treatment when the infestation was high in the control group and remained medium in the treatment group. At the end of the five months, the efficacy of the treatment was 81.8%. As in the present study, no adverse effects were observed. These results are in line with those of the present study and suggest the same effectiveness when the product is used in dogs or cats exposed to environments favorable for flea proliferation.

There is a growing demand by pet owners for so-called “natural” products or methods to protect their pets against parasites. These methods are not checked, and empirical evidence usually shows unsatisfactory efficacy. Relying on plant extracts in antiparasitic protocol may thus improve the regularity of treatment for many owners. On a less subjective note, the wide use of antiparasitic drugs raises questions about the management of wild arthropod and vertebrate populations, given that active molecules are released into the environment, as reported in the professional press [41,42]. This is backed up by scientific evidence and has become a subject of research in ecological studies [43], although it is still in its infancy. The use of less active, more varied compounds may be one way to reduce the environmental impact of such treatments.

The present study has some limitations. Although this study was double-blinded, the number of animals was low, and there were only three sites. The animals were perfectly manageable for flea counting but were much more reluctant to take blood samples. It is regrettable that there is no biological data on the tolerance of the animals to the product. Nevertheless, the data collected in the dog and the clinical follow-up of these cats showed no alterations. The product tested proved to be insufficient for complete control of parasite populations in cats. However, it could be a viable candidate for therapeutic applications involving cats that are rarely exposed, for example, cats that live alone and remain inside and that are not allergic to flea bites. It may also be of value as an alternative to conventional treatments for owners who are looking for a broader range of options. Its use, in combination with existing, more active products, is also potentially relevant, as it could reduce the need for frequent use of more active drugs. For example, one could envisage a protocol based on a year-long supplementation using such a product that is added to food on a regular basis, associated with occasional insecticidal treatment depending on the infestation pressure.

## 4. Material and Methods

### 4.1. Reagents and Chemical Products

The product tested consisted of a proprietary plant blend (Bioticks^®^, Laboratoires Biodevas, Savigné-l’Évêque, France) added to the diet (9 mL/kg) prior to packaging and shipment to the owners. The composition was 37% (*w*/*w*) melissa, 25% (*w*/*w*) thyme, 20% (*w*/*w*) rosemary, 15% (*w*/*w*) absinthe wormwood, 1% (*w*/*w*) lemongrass and fenugreek. The neutral and supplemented feeds were visually identical, save for the letter “A” or “B” marked on the bag. The bags all weighed 5 kg and were stored in an appropriate room in a dry place at a temperature below 25 °C.

Neither the investigators nor the owners were aware of the nature of the feed, and unblinding was performed only after the conclusion of this study.

### 4.2. Animals

Client-owned domestic cats (*Felis catus* Linnaeus, 1758) of mixed or pure breeds in good general health that lived both inside and outside in South-West France (Occitanie region) were included in this study and allocated to either group A or group B.

In addition to good general health, the inclusion criteria included the absence of gastrointestinal, respiratory, or general symptoms and satisfactory results of a normal general physical examination. Mild signs compatible with flea infestation were allowed. No antiparasitic treatment had been administered in the past three months. During the course of this study, no antiparasitic drugs or shampoos were to be used, and treatment of parasites in the environment was not allowed. No other dietary supplement was allowed. The continued use of past treatments that had not been modified during the three months prior to this study, and the use of medication with no known effect on insects and acarids, were allowed.

For the purpose of this study, cats were only fed a standard dry diet specifically formulated for this study. The food was distributed once or twice a day, and the quantities respected the manufacturer’s recommendations to cover the animals’ needs. All the cats had free access to the feed, as well as clean, fresh water at all times. At a given site, all the animals received the same feed. Housing and feeding conditions remained the same throughout this study.

Approval for this study was obtained from the Ethics Committee “*Science et Santé Animales*” (*Animal Science and Health*) N°115 (*École Nationale Vétérinaire de Toulouse*, France) (ref SSA_2020_015). Each owner was given complete information concerning this study prior to enrolment and gave their informed written consent.

### 4.3. Evaluation of Antiparasitic Activity

This study lasted five months (150 days). Fleas were counted before the beginning of this study on day 0 (D0) and, subsequently, on D14, D30, D60, D90, D120, and D150. The recommendations of the European Medication Agency (EMA) for performing flea counts in carnivores in field studies were followed [39]. These recommendations were applicable at the time of this study. Fleas were visually counted following thorough combing of the fur for at least 10 min. If fleas were found during the last five minutes, combing was prolonged for five additional minutes until no flea was found in a five-minute timeframe. All fleas found were counted and removed. Infestation intensity was classified as mild (0–4 fleas per cat), medium (5–9), high (10–19), or very high (20 or more).

### 4.4. Clinical Evaluation of Tolerance

The owner performed immediate and continuous tolerance checks by evaluating the cats’ appetence for the feed and checking for various clinical signs, such as vomiting, diarrhea, decreased appetite, and decreased activity. A general physical examination was performed at each visit by a licensed veterinarian to assess medium-term tolerance. Examinations were only performed on-site. No biological analyses were conducted. An overview is given in Table 2.

### 4.5. Clinical Evaluation of Dermatological Follow-Up

The dermatological evaluation was performed only before this study began (D0) and then on D14, D30, D60, D90, D120, and D150. Lesions recorded were excoriations, alopecia, miliary dermatitis, and eosinophilic plaques. Pruritus was recorded if either seen during the visit or reported by the owner (Table 2).

### 4.6. Statistical Analysis

The efficacy of the product was calculated using Abbott’s formula [44]:

$$\mathrm{Efficacy}\;\left(\%\right)=100\times \frac{\left[\mathrm{mean}\;\mathrm{count}\;\mathrm{in}\;\mathrm{control}\;\mathrm{group}\right]-\left[\mathrm{mean}\;\mathrm{count}\;\mathrm{in}\;\mathrm{treatment}\;\mathrm{group}\right]}{\left[\mathrm{mean}\;\mathrm{count}\;\mathrm{in}\;\mathrm{control}\;\mathrm{group}\right]}$$

Efficacy was calculated in the two groups at each time point, and in each group between D0 and either D14, D30, D60, D90, D120, and D150.

Flea counts were compared with a *t*-test for two independent samples. The significance threshold was set at 0.05. The software used for calculations was XLSTAT, Addinsoft, v. 2020.4.1).

## 5. Conclusions

This prospective, double-blind, placebo-controlled study showed the efficacy of a feed supplemented with Bioticks^®^ in reducing flea populations in healthy cats in the context of five months of continuous use, without the need for any other animal or environmental treatment. Tolerance was very good, and no adverse effect was reported either by the owner or by the veterinarian during clinical examinations.

The use of this product as part of a parasite control protocol, possibly associated with medicine, appears to be a viable option to reduce the use of conventional drugs or for owners seeking plant-based alternatives.

## Figures and Tables

**Table 1:** Flea counts and efficacy calculation (SD: standard deviation; *: significant difference between groups).

		D0	D14	D30	D60	D90	D120	D150
Group A	Count range	11–19	13–16	10–17	10–17	10–20	10–18	10–18
	Mean	14.5	14.4	12.6	13.6	15	14.6	14.3
	SD	2.4	1.0	1.9	2.3	2.6	2.3	2.5
Group B	Count range	14–32	3–25	4–25	4–18	0–13	2–10	1–8
	Mean	21.7	14.8	9.2	10.9	7.2	5	3.3
	SD	5.7	7.5	6.1	5.3	3.9	2.4	2.1
*t*-test *p* value (significance *p* < 0.05)		0.002	0.87	0.112	0.16	<0.0001	<0.0001	<0.0001
		*				*	*	*
Efficacy % treatment vs. placebo		−50%	−3%	27%	20%	52%	66%	77%
Efficacy % placebo (t) vs. D0		/	1%	13%	6%	−3%	−1%	1%
Efficacy % treatment (t) vs. D0		/	32%	58%	50%	67%	77%	85%

**Table 2:** Overview of the different parameters assessed on the animals during this study.

Observer	General Behavior	Appetite	Digestive Signs	Respiratory Signs	Neurologic Signs	Skin Signs	Pruritus
owner	daily observation	daily observation	daily observation	daily observation	daily observation	daily observation	daily observation
investigator	observation during the visit		abdominal palpation	auscultation	observation during the visit	evaluation of alopecia excoriations papules pustules crusts eosinophilic plaques	observation during the visit

## Data Availability

The data presented in this study are available from the corresponding author upon request. The data are not publicly available due to the need to maintain patients’ confidentiality.
